# XIST sponges miR-320d to promote chordoma progression by regulating ARF6

**DOI:** 10.1016/j.jbo.2022.100447

**Published:** 2022-07-16

**Authors:** Yonggang Wang, Zhouzhou Tang, Weichun Guo

**Affiliations:** a Department of Orthopedics, Renmin Hospital of Wuhan University, Wuhan 430060, Hubei Province, China; b Department of Orthopedics, Jingzhou Central Hospital, Jingzhou 434020, Hubei Province, China

**Keywords:** Chordoma, XIST, miR-320d, ARF6

## Abstract

**Background:**

Long non-coding RNAs (lncRNAs) have been demonstrated to play important roles in various tumors, including chordoma. The purpose of this study was to investigate the role and mechanism of lncRNA X-inactive specific transcript (XIST) in chordoma.

**Methods:**

RNA levels and protein levels were measured by real-time quantitative polymerase chain reaction (RT‑qPCR) and western blot assay, respectively. Cell proliferation was assessed by 3-(4,5-dimethylthiazol-2-yl)-2,5-diphenyltetrazolium bromide (MTT) assay, 5-ethynyl-2′-deoxyuridine (EdU) assay and colony formation assay. Tanswell assay was used to examine cell migration and invasion. Cellular glycolysis was examined via the measurement of extracellular acidification rate (ECAR) and lactate production. The interaction between microRNA-320d (miR-320d) and XIST or ADP-ribosylation factor 6 (ARF6) was predicted by bioinformatics analysis and verified by a dual-luciferase reporter and RNA-pull down assays. The xenograft tumor model was used to explore the biological function of XIST *in vivo*.

**Results:**

XIST was overexpressed in chordoma tissues. XIST knockdown suppressed chordoma cell proliferation, migration, invasion, and glycolysis. XIST acted as a sponge of miR-320d. Moreover, miR-320d overexpression inhibited the proliferation, migration, invasion, and glycolysis of chordoma cells. ARF6 was a direct target of miR-320d, and XIST upregulated ARF6 expression via sponging miR-320d. Furthermore, overexpression of ARF6 reversed the inhibitory effects of XIST knockdown on chordoma cell proliferation, migration, invasion, and glycolysis. Importantly, XIST silencing blocked xenograft tumor growth *in vivo*.

**Conclusion:**

XIST knockdown inhibited chordoma progression via regulating the miR-320d/ARF6 axis, providing a novel insight into chordoma pathogenesis.

## Highlights

• XIST was highly expressed in chordoma tissues.
• XIST knockdown inhibited chordoma progression by downregulating ARF6.
• MiR-320d inhibited the malignant behaviors of chordoma cells.
• XIST positively upregulated ARF6 expression via sponging miR-320d in chordoma cells.

## 1 Introduction

Chordoma is a rare primary malignant bone tumor with aggressive and high recurrence [1]. Chordoma is caused by leftover notochordal cells during fetal development and is most common in the sacrum, skull base, and vertebral column [2]. Chordoma is resistant to chemotherapy and poorly sensitive to conventional radiotherapy, so radical surgery combined with adjuvant radiotherapy is the most commonly used treatment [3], [4]. In spite of this, many chordoma patients still suffer local recurrence, with metastasis occurring in 30 % to 40 % of patients, and the overall 5-year postoperative survival rate is about 50 % [5]. Hence, it is critical to clarify the molecular mechanisms that affect the progression of chordoma, which might provide a new theoretical basis for the treatment of chordoma.

Non-coding RNAs (ncRNAs) have gradually become one of the research hotspots in the tumor field in the past few decades [6]. Long non-coding RNAs (lncRNAs; >200 nucleotides) are an important type of ncRNAs without protein-coding capacity [7], [8], [9]. LncRNAs have been demonstrated to play crucial roles in the physiological and pathological processes of various diseases [10], [11]. Moreover, previous studies have shown that lncRNAs are implicated in chordoma tumorigenesis via acting as tumor promoters or suppressors. For example, LOC554202 may play an anti-tumor role in chordoma via suppressing proliferation and invasion of chordoma cells [12]. LncRNA KRT8P41 acted as a tumor promoter via accelerating chordoma cell proliferation and invasion [13]. In addition, the former report indicated that lncRNA X-inactive specific transcript (XIST) was upregulated in chordoma, which might promote the development of chordoma [14]. However, more roles and regulatory mechanisms of XIST in chordoma are still largely unknown.

A large body of evidence has indicated that lncRNAs can upregulate the expression levels of target genes via acting as sponges for microRNAs (miRNAs), thereby affecting cell fate decisions [15], [16]. Dysregulated miRNAs are strongly linked with the development of many diseases, including chordoma [17]. Accumulating evidence has revealed that miR-320d has an anti-tumor role in some tumors, including chordoma [18], [19], [20]. ADP-ribosylation factor 6 (ARF6) is widely expressed in mammalian cells and is used as a tumor-promoting gene in chordoma [21]. By searching the bioinformatics tools, we found that both XIST and ARF6 3′UTR had the complementary binding sequence for miR-320d. According to these findings, we hypothesized that ARF6 might be involved in chordoma development via regulating miR-320d and ARF6.

In our work, we explored XIST expression in chordoma samples. Meanwhile, the role of XIST in chordoma was explored. In addition, the relationships among XIST, miR-320d, and ARF6 in chordoma cells were also investigated using bioinformatic databases and a series of experiments. We aimed to provide a possible therapeutic target for chordoma patients.

## 2 Materials and methods

### 2.1 Specimen collection

In the current study, chordoma tissue specimens (n = 45) and adjacent normal tissue specimens (n = 45) were obtained from chordoma patients who had undergone surgery at Renmin Hospital of Wuhan University. The median age at diagnosis was 65 years old (range: 49–76 years old). All patients had not received any treatment before the operation. The tissues were frozen in liquid nitrogen after resection. Informed consent was obtained from each patient. The detailed clinical characteristics of patients are described in Table 1. This research was approved by the ethics committee of Renmin Hospital of Wuhan University.

**Table 1:** The correlations between lncRNA XIST expression and clinicopathological features of chordoma patients.

Features	n = 45	lncRNA XIST expression		*p*-value
		High(n = 23)	Low(n = 22)	
Age (years)				
<50	14	9	5	0.235
≥50	31	14	17	
Sex				
Male	27	12	15	0.273
Female	18	11	7	
Tumor size (cm)				
<4	25	9	16	0.023*
≥4	20	14	6	
Location				
Superior clivus	24	15	9	0.214
Middle clivus	15	5	10	
Inferior clivus	6	3	3	
Histopathology				
Conventional	35	17	18	0.524
Chondroid	10	6	4	
Invasion condition				
Positive	31	20	11	0.007^**^
Negative	14	3	11	

### 2.2 Cell culture and transfection

Chordoma cells (U-CH1 and JHC7) were purchased from American Type Culture Collection (ATCC; Manassas, VA, USA). U-CH1 cells were grown in a 4:1 mixture of IMDM-RPMI 1640 (ATCC) containing 10 % fetal bovine serum (10 %; Invitrogen, Carlsbad, CA, USA). JHC7 cells were grown in DMEM (ATCC) containing 10 % FBS. All the cells were cultured at 37℃ in humidified air with 5 % CO_2._

Short hairpin RNA (shRNA) targeting XIST (sh-XIST), miR-320d mimic and inhibitor (miR-320d and in-miR-320d), ARF6-overexpression plasmid (ARF6), and their controls (sh-NC, miR-NC, in-miR-NC, and pcDNA) were constructed by RiboBio (Guangzhou, China). The oligonucleotide and plasmid were introduced into chordoma cells (2 × 10^5^ cells/well) by using Lipofectamine 3000 Reagent (Invitrogen).

### 2.3 Real‑time quantitative polymerase chain reaction (RT‑qPCR)

The total RNA was extracted from the tissues (chordoma and normal) and cells (U-CH1 and JHC7) using TRIzol reagent (Invitrogen). Next, cDNA was synthesized using a TIANScript RT Kit (for lncRNA and mRNA; Tiangen Biotech, Beijing, China) or miScript II RT kit (for miRNA; Invitrogen). Thereafter, RT-qPCR reaction was manipulated on a CFX96 Real-Time PCR Detection System (Bio-Rad, Hercules, CA, USA) with SYBR GreenMaster Mix kit (Vazyme, Nanjing, China). 2-^ΔΔCt^ method was used to determine RNA levels. GAPDH and U6 were utilized as the reference controls for lncRNA/mRNA and miRNA, respectively. The primers used in experiments were presented in Table 2.

**Table 2:** Primers sequences used for qRT-PCR.

Name		Primers for qRT-PCR (5′-3′)
lncRNA XIST	Forward	TTACTCTCTCGGGGCTGGAA
	Reverse	AGGGTGTTGGGGGACTAGAA
miR-320d	Forward	GTATGAAAAAGCTGGGTTGAGA
	Reverse	CAGTGCGTGTCGTGGAGT
ARF6	Forward	AACTGGTATGTGCAGCCCTC
	Reverse	GAAAGAGGTGATGGTGGCGA
GAPDH	Forward	AGAAGGCTGGGGCTCATTTG
	Reverse	AGGGGCCATCCACAGTCTTC
U6	Forward	CTCGCTTCGGCAGCACATA
	Reverse	CGAATTTGCGTGTCATCCT

### 2.4 Subcellular localization

PARIS™ Kit (Invitrogen) was utilized for isolating cytoplasm and nucleus fractions. The expression levels of XIST, U6 (a nucleus control), and GAPDH (a cytoplasm control) were determined by RT-qPCR.

### 2.5 Cell proliferation assays

For evaluating the proliferative ability of U-CH1 and JHC7 cells, 3-(4,5-dimethylthiazol-2-yl)-2,5-diphenyltetrazolium bromide (MTT; for detection of cell viability; Beyotime, Jiangsu, China) and EdU (for detection of DNA synthesis; Beyotime) and colony formation assay (for detection of colony-forming ability) assays were performed.

For MTT assay, we seeded U-CH1 and JHC7 cells into a 96-well plate. Each well was added with 5 mg/mL MTT (20 μL) for 4 h at the pointed times. After removing the cultured medium, dimethyl sulfoxide (DMSO; 150 μL; Beyotime) solution was added to each well. At last, a microplate reader (Bio-Rad) was applied to determine the absorbance at 490 nm.

For EdU assay, we seeded U-CH1 and JHC7 cells into 24-well plates. After transfection for 48 h, EdU solution (20 μM) was placed into each well and then incubated for 2 h. Next, cells were fixed using paraformaldehyde (4 %), followed by treatment with Triton-X-100 (0.5 %) to permeabilize the cells. The nucleic acids were stained using DAPI after staining with Click Additive Solution for 0.5 h. Finally, EdU-positive cells were counted and photographed with a fluorescence microscope (Leica, Wetzlar, Germany).

For colony formation assay, we seeded U-CH1 and JHC7 cells into a 6-well plate at a density of 5 × 10^3^ cells/well following transfection for 48 h. Thereafter, these cells were grown in a complete growth medium for 14 days and the culture medium was updated every 2–3 days. Next, cells were subsequently fixed with methanol (Sangon Biotech, Shanghai, China) and stained with 0.4 g/L Giemsa (Beyotime). Lastly, a microscope (Leica) was applied to count the number of colonies (containing ≥ 50 cells per colony).

### 2.6 Transwell assay

Transwell chambers (Costar, Corning, NY, USA) were used for detecting cell migration and invasion. U-CH1 and JHC7 cells were suspended in a serum-free medium and plated in the top chamber pre-coated with (5 × 10^4^ cells for detecting cell invasion) or without (1 × 10^5^ cells for detecting cell migration) Matrigel. Meanwhile, the culture medium with 10 % FBS was placed into the bottom chamber. 24 h later, the non-migrated and non-invaded cells were wiped out with a cotton swab, and 4 % paraformaldehyde (Beyotime) was used to fix the migrated and invaded cells. Next, 0.1 % crystal violet (Beyotime) was used to stain the migrated and invaded cells. Lastly, these cells were photographed by a microscope (Leica).

### 2.7 Extracellular acidification rate (ECAR) assay

To detect ECAR, Seahorse Bioscience XF96 extracellular flux analyzer (Seahorse Bioscience, North Billerica, MA, USA) was used. Briefly, U-CH1 and JHC7 cells were placed in an XF microplate, followed by a baseline measurement. Glucose, Oligomycin (OM; oxidative phosphorylation inhibitor), and 2glycolytic inhibitor (2-DG) were then injected into each well according to the specified time point. At last, the results were analyzed using Seahorse XF96 Wave software (Seahorse Bioscience).

### 2.8 Measurement of lactate production

Based on the manufacturer’s protocol, lactate production was examined using Lactate Assay Kit (BioVision, Milpitas, CA, USA) in U-CH1 and JHC7 cells.

### 2.9 Western blot assay

Total protein was extracted by lysing cells in RIPA lysis buffer (KeyGene, Nanjing, China). After measurement of protein concentration, equal amounts of protein (30 μg per lane) were subjected to 10 % SDS-PAGE. After transferring onto the PVDF membranes (Bio-Rad), the membranes were blocked in 5 % skim milk (Beyotime), followed by immunoblotting with primary antibodies at 4℃for 10–14 h. Next, the corresponding secondary antibody (ab205718; 1:4000; Abcam, Cambridge, UK) was applied in combination with primary antibodies. Lastly, the combined signals were visualized with an enhanced chemiluminescence kit (KeyGene). In this study, the primary antibodies were purchased from Proteintech (Rosemont, IL, USA): E-cadherin (E-cad; 20874–1-AP; 2000), *N*-cadherin (*N*-cad; 22018–1-AP; 1:2000), GLUT1 (21829–1-AP; 1:2000), LDHA (21799–1-AP; 1:5000), ARF6 (20225–1-AP; 1:500) and β-actin (20536–1-AP; 1:2000).

### 2.10 Dual-luciferase reporter assay

Target prediction was administrated through Starbase 3.0 (https://starbase.sysu.edu.cn/). The fragments of XIST and 3′UTR of ARF6 containing the miR-320d binding site region and the corresponding mutated region were synthesized and individually cloned into the psiCHECK2 vector (Promega, Madison, WI, USA), generating XIST-WT/MUT or ARF6 3′UTR WT/MUT. Thereafter, U-CH1 and JHC7 cells were co-introduced with the corresponding reporter vector together with miR-320d/miR-NC for 48 h, followed by detection of the luciferase activity using Lastly, Dual-Luciferase Reporter Gene Assay Kit (Yeasen, Shanghai, China).

### 2.11 RNA pull-down assay

Biotinylated miR-320d (Bio-miR-320d) and biotinylated negative control (Bio-NC) were purchased from RiboBio and introduced into U-CH1 and JHC7 cells for 24 h. After lysing by RIPA buffer (Beyotime), cell extracts were then incubated with M−280 streptavidin magnetic beads (Invitrogen). The RNAs attached to the beads were purified using TRIzol reagent (Invitrogen). Finally, the enrichment of XIST was tested via RT-qPCR after RNA isolation from magnetic beads.

### 2.12 Tumor formation assay

To construct a tumor xenograft model, we purchased BALB/c nude mice from Vital River (Beijing, China). A total of mice (five-week-old; female; n = 10) were segmented into 2 groups (5 mice per group). Approximately 4 × 10^6^ U-CH1 cells (control or XIST knockdown) were inoculated into mice by subcutaneous injection. A caliper was utilized to examine tumor volume every 5 days for 25 days. And we calculated tumor volume using the formula: length × width ^2^ × 1/2. After 25 days, these mice were sacrificed and tumor weight was collected for weighing and further analysis. Animal experiments were conducted with the permission of the Animal Care and Use Committee of Renmin Hospital of Wuhan University. This assay was carried out at least three times.

### 2.13 Immunohistochemistry (IHC) analysis

After fixing in formaldehyde (10 %) and embedding in paraffin, these tissues were then cut into 4-μm-thick sections. Next, these sections were incubated with the Ki67 antibody (1:300; ab16667; Abcam) and cytokeratin (1:250; ab53280; Abcam), followed by incubation with the corresponding secondary antibody (1:2000; ab205718; Abcam). Next, these sections were stained using diaminoaniline (Maxim, Fuzhou, China) and counterstained using haematoxylin (Maxim). Finally, a microscope (Leica) was used to observe these sections.

### 2.14 Statistical analysis

The data were analyzed using GraphPad Prism 7. All data were obtained from at least three repeated experiments and displayed as mean ± standard deviation. The data were analyzed using a Student’s *t*-test or a one-way analysis of variance (ANOVA). The survival curve was analyzed by Kaplan-Meier method. The correlations among XIST, miR-320d and ARF6 were analyzed with Pearson’s correlation coefficient. *P* < 0.05 suggested statistically significant.

## 3 Results

### 3.1 XIST expression was increased in chordoma tissues

**Fig. 1:**
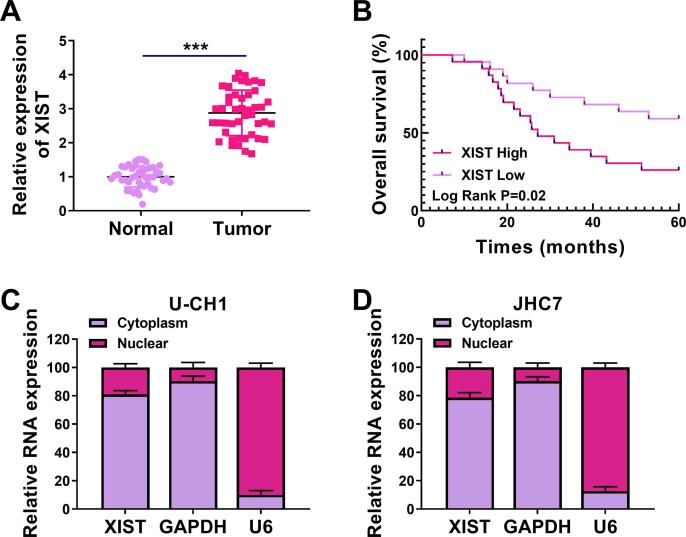
**XIST was upregulated in chordoma tissues.** (A) The expression of XIST was examined by RT-qPCR in chordoma tissues and normal tissues. (B) The overall survival rate was analyzed by Kaplan-Meier analysis in chordoma patients with high and low XIST expression. (C and D) The localization of XIST was determined using RT-qPCR in the nuclear and cytosolic fractions of U-CH1 and JHC7 cells. ***P < 0.001.

To determine XIST expression in chordoma tissues and corresponding adjacent tissues, RT-qPCR was performed. We found that the XIST level was enhanced in chordoma tissues in contrast to normal tissues (Fig. 1A). Moreover, chordoma patients with high XIST expression had a poor overall survival than patients with low XIST expression (Fig. 1B). Meanwhile, our data suggested that XIST expression was associated with Tumor size and Invasion condition (p < 0.05) (Table 1). Next, the localization of XIST was determined in chordoma cells (U-CH1 and JHC7). As shown in Fig. 1C and 1D, XIST was predominantly located in the cytoplasm. These data suggested that XIST might be associated with chordoma progression.

### 3.2 Knockdown of XIST inhibited chordoma cell proliferation, migration, invasion, and glycolysis

**Fig. 2:**
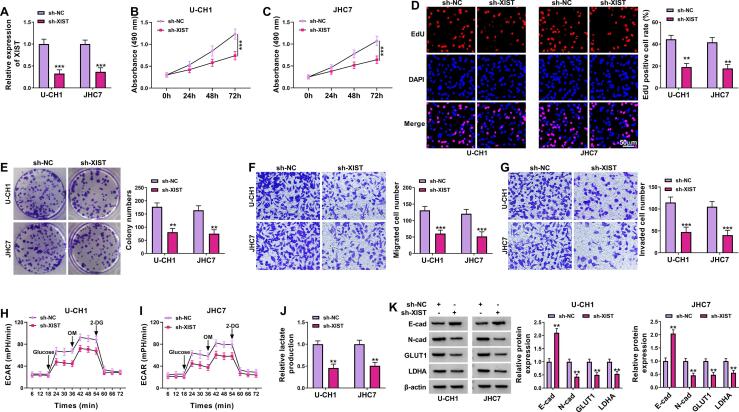
**XIST knockdown inhibited the progression of chordoma cells.** U-CH1 and JHC7 cells were transfected with sh-NC or sh-XIST. (A) The expression of XIST was measured by RT-qPCR. (B and C) MTT assay was used to assess cell viability. (D) DNA synthesis was assessed using EdU assay. (E) Colony formation assay was performed to detect the number of colonies. (F and G) Transwell assay was applied to assess cell migration and invasion. (H and I) Seahorse Bioscience XF96 extracellular flux analyzer was utilized to measure ECAR. (J) Lactate production was assessed using Lactate Assay kit. (K) Western blot assay was performed to analyze the protein levels of E-cad, *N*-cad, GLUT1, and LDHA. **P < 0.01, ***P < 0.001.

To investigate the biological role of XIST in chordoma cells, we performed loss-of-function experiments by transfection of shRNA in U-CH1 and JHC7 cells. Transfection of sh-XIST resulted in a significant downregulation of XIST expression in U-CH1 and JHC7 cells (Fig. 2A). MTT assay, EdU assay, and colony formation assay showed that XIST silencing repressed cell viability, DNA synthesis, and colony formation ability in U-CH1 and JH7C cells, indicating that XIST knockdown inhibited chordoma cell proliferation (Fig. 2B-2E). Transwell assay indicated that U-CH1 and JHC7 cell migration and invasion were suppressed after downregulating XIST (Fig. 2F and 2G). Next, we explored whether XIST played a critical role in glycolysis in chordoma cells. The ECAR approximated the glycolysis fux rate. We observed a decrease in ECAR in U-CH1 and JHC7 cells transfected with sh-XIST (Fig. 2H and 2I). Moreover, XIST silencing significantly decreased lactate production (Fig. 2J). Western blot assay indicated that E-cad (an epithelial marker) protein level was increased and *N*-cad (a mesenchymal marker), GLUT1 and LDHA (glycolysis markers) protein levels were reduced after knockdown of XIST (Fig. 2K). All these data indicated that XIST knockdown inhibited the progression of chordoma cells.

### 3.3 MiR-320d was a direct target of XIST

**Fig. 3:**
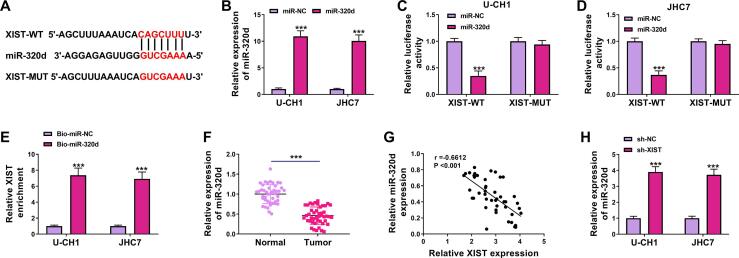
**XIST directly interacted with miR-320d.** (A) Predicted binding sites between miR-320d and XIST were shown. (B) The expression of miR-320d was detected by RT-qPCR in U-CH1 and JHC7 cells transfected with miR-NC or miR-320d. (C and D) Dual-luciferase reporter assay was performed to examine the luciferase activity in U-CH1 and JHC7 cells co-transfected with miR-NC or miR-320d and XIST-WT or XIST-MUT. (E) The level of XIST was examined by RNA pull-down assay in U-CH1 and JHC7 cells transfected with Bio-miR-NC or Bio-miR-320d. (F) The expression of miR-320d was detected by RT-qPCR in chordoma tissues and normal tissues. (G) The correlation between XIST and miR-320d in chordoma tissues was analyzed. (H) The level of miR-320d was assessed by RT-qPCR in U-CH1 and JHC7 cells transfected with sh-NC or sh-XIST. ***P < 0.001.

LncRNAs have been increasingly reported to regulate the expression of target genes via sponging certain miRNAs. Next, we used an online bioinformatics database starBase to predict the potential targets of XIST. As presented in Fig. 3A, miR-320d had putative binding sites for XIST. Transfection of miR-320d increased the expression of miR-320d in U-CH1 and JHC7 cells (Fig. 3B), indicating a high transfection efficiency. Dual-luciferase reporter and RNA pull-down assays were performed to further confirm whether XIST could directly bind to miR-320d. The results demonstrated that miR-320d overexpression strikingly reduced the luciferase activity of XIST-WT but not XIST-MUT (Fig. 3C and 3D). Meanwhile, the enrichment of XIST was markedly increased in the Bio-miR-320d group (Fig. 3E). In addition, miR-320d was found to be downregulated in chordoma tissues compared to normal tissues (Fig. 3F). A negative correlation between miR-320d and XIST expression was observed in chordoma tissues (Fig. 3G). Next, the effect of XIST on miR-320d expression was explored. Knockdown of XIST increased miR-320d expression (Fig. 3H). Taken together, these findings demonstrated that miR-320d was targeted by XIST.

### 3.4 Overexpression of miR-320d suppressed the proliferation, migration, invasion, and glycolysis in chordoma cells

**Fig. 4:**
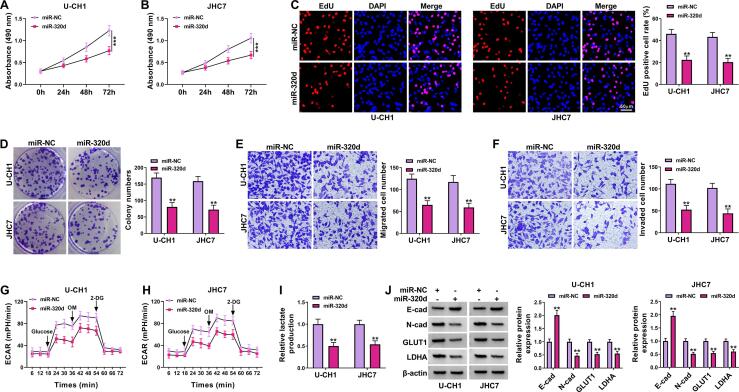
**MiR-320d exerted an anti-tumor role in chordoma cells.** U-CH1 and JHC7 cells were transfected with miR-NC or miR-320d. (A-D) Cell proliferation was assessed by MTT assay, EdU assay and colony formation assay. (E and F) Cell migration and invasion were examined using transwell assay. (G and H) ECAR was detected using a Seahorse Bioscience XF96 extracellular flux analyzer. (I) Lactate production was evaluated by Lactate Assay kit. (J) The protein levels of E-cad, *N*-cad, GLUT1, and LDHA were determine by western blot assay. **P < 0.01, ***P < 0.001.

To detect the role of miR-320d in chordoma cells, we overexpressed miR-320d expression in chordoma cells. We found that miR-320d upregulation suppressed cell viability, DNA synthesis, colony-forming ability, migration, and invasion in U-CH1 and JHC7 cells (Fig. 4A-4F). Moreover, ECAR and lactate production was decreased by overexpression of miR-320d in U-CH1 and JHC7 cells (Fig. 4G-4I). Further, the upregulation of miR-320d increased E-cad protein expression and reduced the protein levels of *N*-cad, GLUT1, and LDHA in U-CH1 and JHC7 cells (Fig. 4J). Collectively, miR-320d might play an anti-tumor role in chordoma.

### 3.5 ARF6 was targeted by miR-320d in chordoma cells

**Fig. 5:**
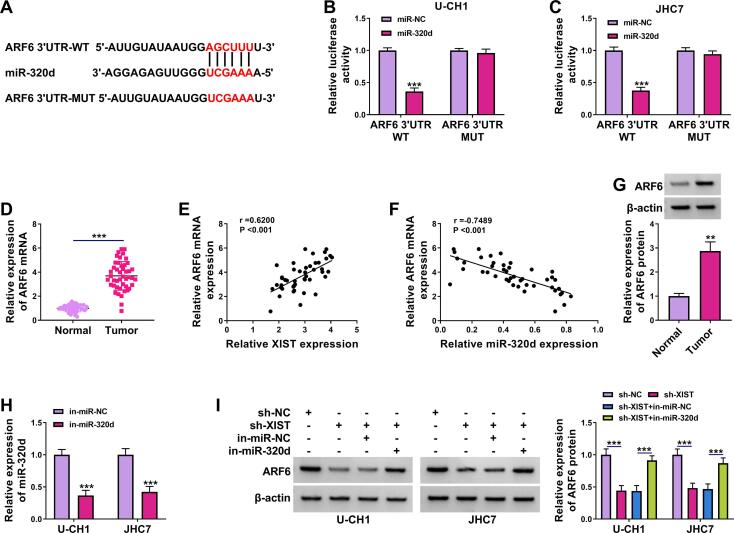
**ARF6 was directly targeted by miR-320d.** (A) The putative binding sites between miR-320d and ARF6 3′UTR were predicted by starBase. (B and C) The interaction between miR-320d and ARF6 was confirmed using dual-luciferase reporter assay in U-CH1 and JHC7 cells. (D) The expression of ARF6 mRNA was detect using RT-qPCR in chordoma tissues and normal tissues. (E and F) The correlation between ARF6 mRNA expression and miR-320d or XIST expression in chordoma tissues was analyzed. (G) The expression of ARF6 protein was examined by western blot assay in chordoma tissues and normal tissues. (H) The expression of miR-320d was tested using RT-qPCR in U-CH1 and JHC7 cells transfected with in-miR-NC or in-miR-320d. (I) ARF6 protein expression was measured by western blot assay in U-CH1 and JHC7 cells transfected with sh-NC, sh-XIST, sh-XIST + in-miR-NC, or sh-XIST + in-miR-320d. **P < 0.01, ***P < 0.001.

As most miRNAs can modulate the expression of their downstream target genes to exert their biological functions, we used starBase to predict the target of miR-320d. We observed that 3′UTR of ARF6 shared binding sites for miR-320d (Fig. 5A), suggesting that ARF6 might be a target for miR-320d. The results of the dual-luciferase reporter assay showed that miR-320d overexpression markedly reduced the luciferase activity of ARF6 3′UTR WT, but did not affect the luciferase activity of ARF6 3′UTR MUT in U-CH1 and JHC7 cells (Fig. 5B and 5C). Then, we explored ARF6 mRNA expression in chordoma tissues, and the data suggested that ARF6 mRNA expression was higher in chordoma tissues than that in normal tissues, and its expression was negatively correlated with miR-320d expression and positively correlated with XIST expression in chordoma tissues (Fig. 5D-5F). ARF6 protein expression was also increased in chordoma tissues (Fig. 5G). Transfection of in-miR-320d markedly reduced miR-320d expression in U-CH1 and JHC7 cells (Fig. 5H), suggesting that miR-320d was successfully inhibited. Next, we explored whether XIST regulated ARF6 expression by sponging miR-320d. The data showed that XIST silencing reduced the protein expression of ARF6, which was reversed by downregulating miR-320d (Fig. 5I), indicating that XIST sponged miR-320d to positively regulate ARF6 expression.

### 3.6 XIST silencing repressed malignancy of chordoma cells by regulating ARF6

**Fig. 6:**
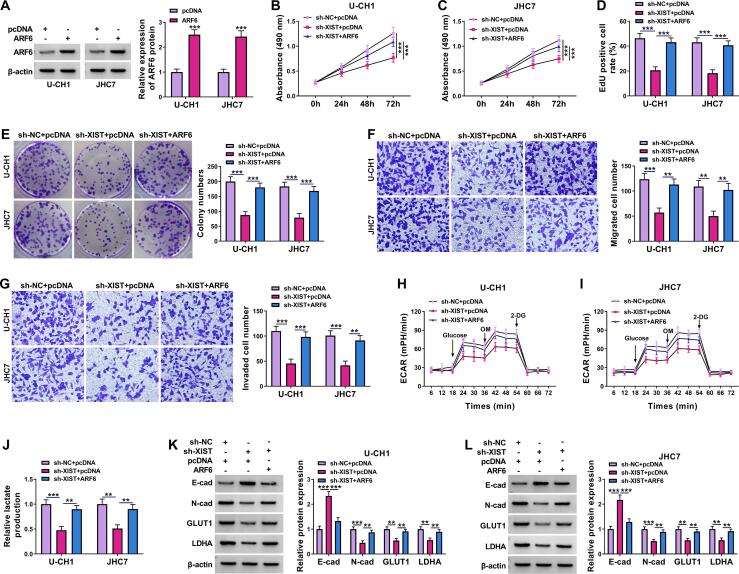
**Knockdown of XIST suppressed chordoma cell proliferation, metastasis and glycolysis via reducing ARF6 expression.** (A) ARF6 protein expression was determined by western blot assay in U-CH1 and JHC7 cells transfected with pcDNA or ARF6. (B-L) U-CH1 and JHC7 cells were transfected with sh-NC + pcDNA, sh-XIST + pcDNA or sh-XIST + ARF6. (B-E) MTT assay, EdU assay and colony formation assay were used to measure cell proliferation. (F and G) Cell migration and invasion were measured using transwell assay. (H and I) ECAR was examined by Seahorse Bioscience XF96 extracellular flux analyzer. (J) Lactate Assay kit was used to measure lactate production. (K and L) E-cad, *N*-cad, GLUT1, and LDHA protein expression were detected using western blot assay. **P < 0.01, ***P < 0.001.

Transfection of ARF6 increased ARF6 protein expression in U-CH1 and JHC7 cells (Fig. 6A), suggesting a high transfection efficiency. To determine whether XIST regulated chordoma cell behaviors by targeting ARF6, we performed rescue experiments in U-CH1 and JHC7 cells. The inhibitory effects of XIST knockdown on cell viability, DNA synthesis, colony-forming ability, migration, and invasion were overturned by the addition of ARF6 (Fig. 6B-6G). Moreover, ARF6 overexpression counteracted the suppressive effects of XIST downregulation on ECAR and lactate production in U-CH1 and JHC7 cells (Fig. 6H-6 J). In addition, the upregulation of E-cad protein expression and the downregulation of *N*-cad, GLUT1, and LDHA protein expression caused by XIST silencing was reversed by increasing ARF6 expression (Fig. 6K and 6L). Overall, these results suggested that ARF6 overexpression abated inhibitory effects of XIST downregulation on chordoma cell proliferation, metastasis, and glycolysis.

### 3.7 XIST downregulation inhibited chordoma tumorigenicity *in vivo*

**Fig. 7:**
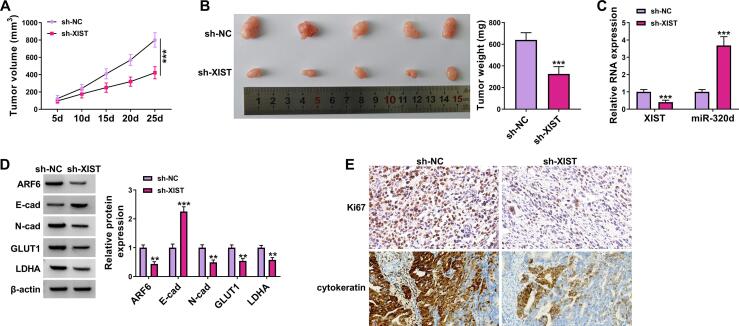
**XIST silencing blocked the tumorigenesis of chordoma *in vivo*.** U-CH1 cells were introduced into nude mice to establish xenograft tumor model. (A) Tumor volume was measured at the indicated time points (5d, 10d, 15d, 20d, and 25d). (B) At 25d upon cell implantation, tumors were excised, imaged, and weighted. (C) XIST and miR-320d expression were measured by RT-qPCR in excised tumor tissues. (D) Western blot assay was applied to analyze the protein levels of ARF6, E-cad, *N*-cad, GLUT1, and LDHA. (E) IHC analysis was used to detect Ki67 and cytokeratin expression in tumor tissues. **P < 0.01, ***P < 0.001.

Next, we explored whether XIST acted as a tumor promoter *in vivo.* Significant inhibition of volume and weight was observed after XIST knockdown (Fig. 7A and 7B). The results of RT-qPCR showed that XIST knockdown reduced the level of XIST and enhanced miR-320d abundance in tumor tissues (Fig. 7C). XIST knockdown inhibited ARF6, *N*-cad, GLUT1, and LDHA protein expression and increased E-cad protein expression in tumor tissues (Fig. 7D). IHC analysis showed that XIST interference suppressed Ki67 (a proliferation marker) and cytokeratin (a chordoma marker) expression in tumor tissues (Fig. 7E). Collectively, these *in vivo* data indicated that XIST downregulation could inhibit the tumorigenicity of chordoma.

## 4 Discussion

The previous study showed that some lncRNAs are dysregulated in human chordoma, and they might help improve the diagnosis and treatment of chordoma [22], [23]. In this work, we demonstrated that XIST was highly expressed in chordoma, and its silencing limited chordoma cell proliferation, metastasis, and glycolysis via regulating the miR-320d/ARF6 axis.

Many studies revealed that abnormal expression of XIST was related to the progression and development of diverse tumors. For example, XIST expression was upregulated in colorectal cancer, and its downregulation repressed colorectal cancer cell proliferation, migration, and invasion [24]. Moreover, XIST acted as an upregulated lncRNA in liver cancer to promote liver cancer cell growth and metastasis [25]. In addition, XIST could also facilitate the migration and invasion of papillary thyroid cancer cells [26]. By contrast, downregulation of XIST was observed in ovarian cancer cells and tissues, and XIST suppressed ovarian cancer cell proliferation and inversely promoted cell apoptosis [27]. Further, XIST overexpression hampered breast cancer cell progression *in vitro* and *in vivo* [28]. These results indicated that XIST played a different role in different cancers. In chordoma, XIST expression is closely correlated with the poor prognosis of the patient, and XIST promoted chordoma cell proliferation and inhibited apoptosis [14]. In our research, XIST upregulation was also observed in chordoma tissues. XIST silencing repressed chordoma cell proliferation, migration, invasion, and glycolysis. Importantly, XIST downregulation inhibited the tumorigenesis of chordoma *in vivo*. These results disclosed that XIST might act as a tumor facilitator in chordoma.

In recent years, the interaction between miRNAs and lncRNAs has become one of the research hotspots in the study of tumor pathological mechanisms. Given that lncRNAs in the cytoplasm can function as miRNA sponges via interacting with miRNAs to positively regulate the expression and function of downstream target mRNAs [29]. Here, XIST was predominantly located in the cytoplasm. Hence, we assumed that XIST might absorb miRNA. As expected, XIST was a sponge for miR-320d. Previous studies have indicated that miR-320d served as a tumor suppression miRNA in some cancers, and its downregulation is closely related to the poor prognosis of patients [30], [31]. In chordoma, miR-320d was lowly expressed, and miR-320d inhibition increased the growth, invasion, and migration of chordoma cells [20]. Consistently, we also uncovered that miR-320d content was declined in chordoma tissues. Functional experiments showed that miR-320d upregulation could restrict the growth, metastasis, and glycolysis of chordoma cells, suggesting that miR-320d has an anti-tumor role in chordoma.

MiRNAs can bind to target mRNAs to regulate many biological and pathological processes [32]. Next, we analyzed the downstream target of miR-320d. Our data proved that ARF6 could be targeted by miR-320d. ARF6 is a member of the ARF protein family of small GTPases, which plays a critical role in pathological and physiological processes [33], [34]. In addition, the available evidence indicated that ARF6 acted as an oncogene in many cancers to promote the proliferation invasion, and migration of cancer cells [35], [36]. ARF6 is also believed to be implicated in the glycolysis of cancer cells [37]. Importantly, an increase in ARF6 expression was found in chordoma tissues, and ARF6 overexpression reversed the suppressive effect of MDFIC-7 silencing on proliferation and aerobic glycolysis of chordoma cells [21]. Here, we also observed upregulation of the ARF6 expression in chordoma tissue samples. Overexpression of ARF6 could abate the suppressive influence of XIST deficiency on chordoma cell growth, metastasis, and glycolysis. Mechanistically, XIST positively modulated the ARF6 level via sponging miR-320d. Compared with previous articles [14], the novelty of this study lies in the confirmation that XIST downregulation might inhibit tumor migration, invasion, and glycolysis, and the construction of a novel network mechanism of the XIST-miR-320d-ARF6 axis. These data indicated that XIST served as a sponge of miRNAs to regulate the more comprehensive lncRNA-miRNA-mRNA co-expression network.

In conclusion, our results proved that XIST knockdown suppressed chordoma cell growth, metastasis, and glycolysis as well as inhibited tumor growth *in vivo* by regulating the miR-320d/ARF6 axis. This is the first report to demonstrate that the XIST miR-320d/ARF6 axis contributed to the tumorigenesis of human chordoma. These results might offer potential targets for chordoma therapy and help understand the mechanisms of chordoma progression.

## Declaration of Competing Interest

The authors declare that they have no known competing financial interests or personal relationships that could have appeared to influence the work reported in this paper.

## References

[1] Gulluoglu S., Turksoy O., Kuskucu A., Ture U., Bayrak O.F. (2016). The molecular aspects of chordoma. Neurosurg Rev 39(2).

[2] Walcott B.P., Nahed B.V., Mohyeldin A., Coumans J.V., Kahle K.T., Ferreira M.J. (2012). Chordoma: current concepts, management, and future directions. Lancet Oncol.

[3] Pamir M.N., Ozduman K. (2008). Tumor-biology and current treatment of skull-base chordomas. Adv Tech Stand Neurosurg.

[4] Kayani B., Hanna S.A., Sewell M.D., Saifuddin A., Molloy S., Briggs T.W. (2014). A review of the surgical management of sacral chordoma. Eur J Surg Oncol.

[5] Wedekind M.F., Widemann B.C., Cote G. (2021). Chordoma: Current status, problems, and future directions. Curr Probl Cancer.

[6] Anastasiadou E., Jacob L.S., Slack F.J. (2018). Non-coding RNA networks in cancer. Nat Rev Cancer.

[7] Li J., Xuan Z., Liu C. (2013). Long Non-Coding RNAs and Complex Human Diseases. Int J Mol Sci.

[8] Gibb E.A., Brown C.J., Lam W.L. (2011). The functional role of long non-coding RNA in human carcinomas. Mol Cancer.

[9] Zhang X., Sun S., Pu J.K.S., Tsang A.C.O., Lee D., Man V.O.Y., Lui W.M., Wong S.T.S., Leung G.K.K. (2012). Long non-coding RNA expression profiles predict clinical phenotypes in glioma. Neurobiol Dis.

[10] Zhou Z., Zhu Y., Gao G., Zhang Y. (2019). Long noncoding RNA SNHG16 targets miR-146a-5p/CCL5 to regulate LPS-induced WI-38 cell apoptosis and inflammation in acute pneumonia. Life Sci.

[11] Ghafouri-Fard S., Shoorei H., Taheri M. (2020). Non-coding RNAs are involved in the response to oxidative stress. Biomed Pharmacother.

[12] Ma X., Qi S., Duan Z., Liao H., Yang B., Wang W., Tan J., Li Q., Xia X. (2017). Long non-coding RNA LOC554202 modulates chordoma cell proliferation and invasion by recruiting EZH2 and regulating miR-31 expression. Cell Prolif.

[13] Wen H., Fu Y., Zhu Y., Tao S., Shang X., Li Z., You T., Zhang W. (2021). Long non-coding RNA KRT8P41/miR-193a-3p/FUBP1 axis modulates the proliferation and invasion of chordoma cells. J Bone Oncol.

[14] Hai B., Pan X., Du C., Mao T., Jia F., Liu Y., Ma Y., Liu X., Zhu B. (2020). LncRNA XIST Promotes Growth of Human Chordoma Cells by Regulating miR-124-3p/iASPP Pathway. Onco Targets Ther.

[15] Salmena L., Poliseno L., Tay Y., Kats L., Pandolfi P.P. (2011). A ceRNA hypothesis: the Rosetta Stone of a hidden RNA language?. Cell.

[16] Dong W., Li J., Dong X., Shi W., Zhang Y., Liu Y. (2021). MiR-17 and miR-93 Promote Tumor Progression by Targeting p21 in Patients with Chordoma. Onco Targets Ther.

[17] Kwan J.Y., Psarianos P., Bruce J.P., Yip K.W., Liu F.F. (2016). The complexity of microRNAs in human cancer. J Radiat Res 57 Suppl.

[18] Qin C.Z., Lv Q.L., Yang Y.T., Zhang J.M., Zhang X.J., Zhou H.H. (2017). Downregulation of MicroRNA-320d predicts poor overall survival and promotes the growth and invasive abilities in glioma. Chem Biol Drug Des.

[19] Liu X., Xu X., Pan B., He B., Chen X., Zeng K., Xu M., Pan Y., Sun H., Xu T., Hu X., Wang S. (2019). Circulating miR-1290 and miR-320d as Novel Diagnostic Biomarkers of Human Colorectal Cancer. J Cancer.

[20] Zhang K., Liu Z., Tang Y., Shao X., Hua X., Liu H., Yang H., Chen K. (2021). LncRNA NONHSAT114552 Sponges miR-320d to Promote Proliferation and Invasion of Chordoma Through Upregulating NRP1. Front Pharmacol.

[21] Zhang K., Liu Z., Wang Z., Zhou Z., Shao X., Hua X., Mao H., Yang H., Ren K., Chen K. (2021). Long Non-Coding RNA MDFIC-7 Promotes Chordoma Progression Through Modulating the miR-525-5p/ARF6 Axis. Front Oncol.

[22] Wang B., Zhang K., Meng S., Shao X., Zhou Z., Mao H., Zhu Z., Chen H., Yang H., Chen K. (2021). LncRNA-NONHSAT024778 promote the proliferation and invasion of chordoma cell by regulating miR-1290/Robo1 axis. Int J Biol Sci.

[23] Gong F., Wang X., Sun Q., Su X., Hu X., Liu B. (2021). Long non-coding RNA LINC00525 interacts with miR-31-5p and miR-125a-5p to act as an oncogenic molecule in spinal chordoma. Biochem Biophys Res Commun.

[24] Li W., He Y., Cheng Z. (2021). Long noncoding RNA XIST knockdown suppresses the growth of colorectal cancer cells via regulating microRNA-338-3p/PAX5 axis. Eur J Cancer Prev.

[25] Liu L., Jiang H., Pan H., Zhu X. (2021). LncRNA XIST promotes liver cancer progression by acting as a molecular sponge of miR-200b-3p to regulate ZEB1/2 expression. J Int Med Res.

[26] Du Y.L., Liang Y., Cao Y., Liu L., Li J., Shi G.Q. (2021). LncRNA XIST Promotes Migration and Invasion of Papillary Thyroid Cancer Cell by Modulating MiR-101-3p/CLDN1 Axis. Biochem Genet.

[27] Guo T., Yuan D., Zhang W., Zhu D., Xiao A., Mao G., Jiang W., Lin M., Wang J. (2021). Upregulation of long noncoding RNA XIST has anticancer effects on ovarian cancer through sponging miR-106a. Hum Cell.

[28] Liu B., Luo C., Lin H., Ji X., Zhang E., Li X. (2021). Long Noncoding RNA XIST Acts as a ceRNA of miR-362-5p to Suppress Breast Cancer Progression. Cancer Biother Radiopharm.

[29] J.-H. Yoon, K. Abdelmohsen, M. Gorospe, editors. Functional interactions among microRNAs and long noncoding RNAs. Semin Cell Dev Biol; 2014: Elsevier. 10.1016/j.semcdb.2014.05.015 PMC4163095 24965208

[30] Yufeng Z., Ming Q., Dandan W. (2021). MiR-320d Inhibits Progression of EGFR-Positive Colorectal Cancer by Targeting TUSC3. Front Genet.

[31] Li W., Ding X., Wang S., Xu L., Yin T., Han S., Geng J., Sun W. (2020). Downregulation of serum exosomal miR-320d predicts poor prognosis in hepatocellular carcinoma. J Clin Lab Anal.

[32] Felekkis K., Touvana E., Stefanou C., Deltas C. (2010). microRNAs: a newly described class of encoded molecules that play a role in health and disease. Hippokratia.

[33] Sabe H. (2003). Requirement for Arf6 in cell adhesion, migration, and cancer cell invasion. J Biochem.

[34] Li R., Peng C., Zhang X., Wu Y., Pan S., Xiao Y. (2017). Roles of Arf6 in cancer cell invasion, metastasis and proliferation. Life Sci.

[35] Zaoui K., Rajadurai C.V., Duhamel S., Park M. (2019). Arf6 regulates RhoB subcellular localization to control cancer cell invasion. J Cell Biol.

[36] Hashimoto S., Furukawa S., Hashimoto A., Tsutaho A., Fukao A., Sakamura Y., Parajuli G., Onodera Y., Otsuka Y., Handa H., Oikawa T., Hata S., Nishikawa Y., Mizukami Y., Kodama Y., Murakami M., Fujiwara T., Hirano S., Sabe H. (2019). ARF6 and AMAP1 are major targets of KRAS and TP53 mutations to promote invasion, PD-L1 dynamics, and immune evasion of pancreatic cancer. Proc Natl Acad Sci U S A.

[37] Liang C., Qin Y., Zhang B., Ji S., Shi S., Xu W., Liu J., Xiang J., Liang D., Hu Q., Ni Q., Yu X., Xu J. (2017). ARF6, induced by mutant Kras, promotes proliferation and Warburg effect in pancreatic cancer. Cancer Lett.

